# Pharmacogenetic study of seven polymorphisms in three nicotinic acetylcholine receptor subunits in smoking-cessation therapies

**DOI:** 10.1038/s41598-017-16946-6

**Published:** 2017-12-01

**Authors:** Giulia Pintarelli, Antonella Galvan, Paolo Pozzi, Sara Noci, Giovanna Pasetti, Francesca Sala, Ugo Pastorino, Roberto Boffi, Francesca Colombo

**Affiliations:** 1 Department of Research, Fondazione IRCCS Istituto Nazionale dei Tumori, Milan, Italy; 2 Department of Surgery, Fondazione IRCCS Istituto Nazionale dei Tumori, Milan, Italy; 3 Tobacco Control Unit, Fondazione IRCCS Istituto Nazionale dei Tumori, Milan, Italy; 4 Department of Predictive and Preventive Medicine, Fondazione IRCCS Istituto Nazionale dei Tumori, Milan, Italy; 5 Cardiopulmonary Rehabilitation Unit, Azienda Sociosanitaria Territoriale Lariana, Sant’Antonio Abate Hospital, Cantù (CO), Italy

## Abstract

Smoking-cessation therapy reduces the risk of smoking-related diseases, but is successful only in a fraction of smokers. There is growing evidence that genetic variations in nicotinic acetylcholine receptor (nAChR) subunits influence the risk of nicotine dependence and the ability to quit smoking. To investigate the role of polymorphisms in nAChR genes on smoking quantity and the outcome of smoking-cessation therapies, we carried out an association study on 337 smokers who underwent pharmacotherapy with varenicline, bupropion, nicotine replacement therapy (NRT) alone, or NRT plus bupropion. Smoking habit and abstention were assessed from the number of cigarettes smoked per day (CPD) and the exhaled CO (eCO), at baseline and up to 12 months. We genotyped seven polymorphisms in genes encoding the nAChR subunits *CHRNA4*, *CHRNA5*, and *CHRNB2*. At baseline, both CPD and eCO were associated with polymorphisms in the *CHRNA5* locus (rs503464, rs55853698, rs55781567 and rs16969968; *P* < 0.01). rs503464, a variant in the 5′-UTR of *CHRNA5*, was also associated with short-, mid- and long-term responses to therapy (*P* = 0.011, *P* = 0.0043, *P* = 0.020, respectively), although after correction for multiple testing only the association at the mid-term assessment remained significant (FDR = 0.03). These data support the role of individual genetic makeup in the ability to quit smoking.

## Introduction

Cigarette smoking is the leading cause of avoidable morbidity and mortality in the world^1^. Tobacco use increases the risk of death from many common diseases, including cardiovascular and non-neoplastic pulmonary diseases as well as different cancers^2,3^. Quitting smoking before the age of 40 reduces the risk of dying from smoking-related disease by about 90%^3,4^. However, quitting is difficult, as approximately only 6% of smokers manage to quit on their own annually^5^.

The ability to quit smoking is negatively influenced by nicotine dependence, as heavy smokers are more likely to fail to cease smoking than light smokers^6^. Genetic studies have widely demonstrated that smoking cessation and nicotine addiction are genetically determined^7^, and several chromosomal loci have been associated with these phenotypes^8–12^. Of note, three such loci contain clusters of genes coding for six nicotinic acetylcholine receptor (nAChR) subunits: *CHRNB3-CHRNA6* on chromosome 8p11, *CHRNA5-CHRNA3-CHRNB4* on chromosome 15q25, and *CHRNA4* on chromosome 20q13. Single nucleotide variations (SNPs) in these loci have repeatedly been shown to be significantly associated with nicotine dependence (reviewed in^13^). SNPs in another nAChR subunit, *CHRNB2* on chromosome 1, are also believed to associate with nicotine dependence^14^.

The nAChRs are cation channels activated by acetylcholine and expressed in the nervous system, muscles and lungs. These receptors are pentameric proteins composed of various combinations of subunits named α1–10, β1–4, γ δ and ε. nAChRs bear the prime responsibility for tobacco addiction^15^, since they are also activated by nicotine, the cigarette’s major biologically active substance^16^. nAChRs are therefore targets of smoking-cessation therapies, the most effective of which are nicotine replacement therapy (NRT), varenicline, and bupropion^17^. Varenicline binds α4β2-containing nAChR receptors as a partial agonist^18^, while bupropion is a noncompetitive antagonist of nAChRs^19^. The efficacy of these treatments is still limited and variable^20^. This variability could be due to genetic differences in the nAChRs, as several pharmacogenetic studies have found significant associations between genetic polymorphisms in nAChR subunit genes and the success of pharmacological smoking-cessation treatments (reviewed in^12,21^). More recently, King *et al*.^12^ found associations between abstinence during varenicline treatment and SNPs in *CHNRA4*, *CHNRA5* (and other genes in the chromosome 15q25 locus) and *CHNRB2*, but no association between abstinence under bupropion treatment and nAChR SNPs. Bergen *et al*.^22^ reported associations between NRT success and two SNPs in the chromosome 15q25 locus, but not with a SNP in *CHNRB2*.

To further evaluate the possibility that genetic variations in nAChR subunits influence smoking habit and the effectiveness of smoking-cessation therapies, we studied a cohort of adult smokers who sought pharmacological treatment in our institute. For the present study, we genotyped seven selected SNPs in three nAChR subunits. In *CHNRA4*, we studied rs2236196, previously associated with abstinence during varenicline treatment^12^ but not with smoking cessation during NRT^23^. In *CHRNA5*, we investigated five SNPs, including the non-synonymous coding variant rs16969968, suggested to affect the risk of nicotine dependence by altering the function of α5-containing nAChRs^24^, as well as three SNPs in the 5′-UTR (rs503464, rs55853698, and rs55781567) and one 22-bp insertion/deletion in the promoter (rs3841324), all suggested to be involved in nicotine dependence by modulating *CHRNA5* subunit mRNA levels^25–30^. Finally, in *CHRNB2*, we genotyped rs2072661, previously associated with nicotine dependence^14^ but not with response to pharmacological or behavioural therapies for smoking cessation^22^. The overall aim of our study was to identify genetic variants that can predict the intensity of the smoking habit and the effectiveness of pharmacological smoking-cessation therapies.

## Results

The study considered 337 Italian adults (197 men and 140 women) who participated in smoking-cessation programs (Table 1). Before beginning treatment, they consumed a median of 20 cigarettes per day (CPD) and had a median expired CO level (eCO) of 20 parts per million (ppm). Two thirds of participants had received varenicline, while the others were treated with bupropion, NRT, or both.

**Table 1:** Characteristics of 337 Italian subjects treated with smoking-cessation drugs.

Characteristic	N (%) or median (range)
Sex, n (%)	
*Male*	197 (58)
*Female*	140 (42)
Age at therapy start, years	55 (19–75)
Baseline cigarettes per day, n	20 (1–70)
Baseline eCO, ppm	20 (0–75)
Therapy, n (%)	
Varenicline	225 (66.7)
Bupropion	34 (10.2)
NRT	67 (19.8)
Bupropion + NRT	11 (3.3)

eCO = expired carbon monoxide; NRT = nicotine replacement therapy.

**Figure 1:**
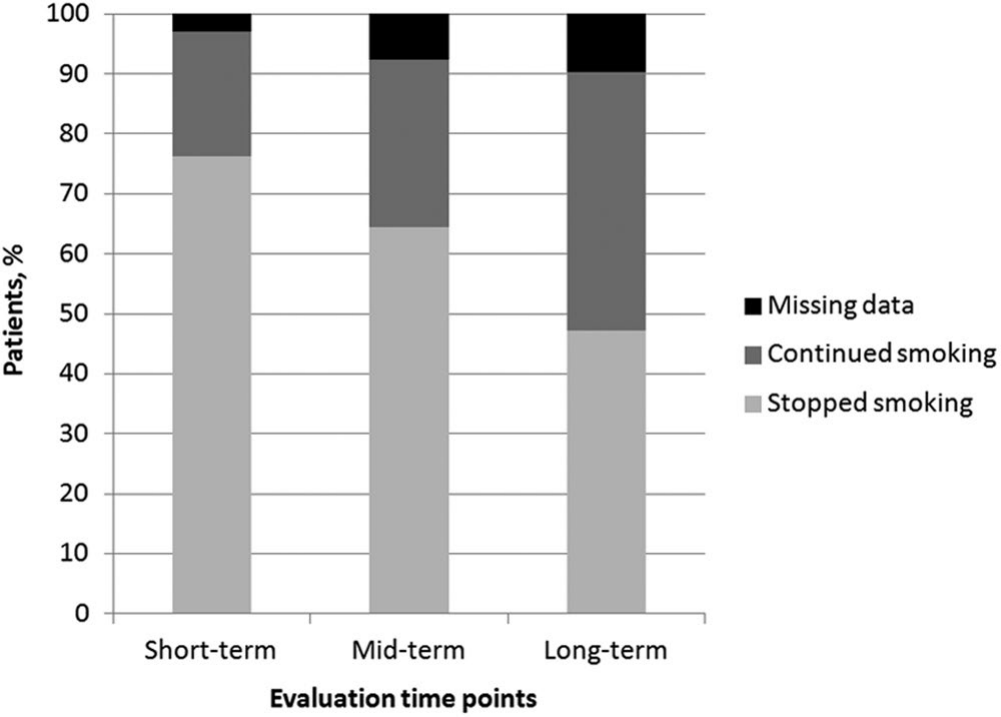
Response to smoking-cessation therapies, for 337 smokers, at the short-, mid- and long-term follow-up examinations (1, 3, and 12 months after the start of treatment, respectively).

One month after the start of treatment, at the short-term evaluation, the majority of patients (76.3%) had stopped smoking (Fig. 1). At the mid- and long-term evaluations, corresponding to three and 12 months after the start of treatment, the percentage of patients who did not smoke had decreased to 64.4% and 47.2%, respectively, due to smoking relapse. Indeed, 92 patients who had quit at the short-term follow-up restarted smoking within one year of the start of treatment. There was no association between the type of therapy and the success of quitting and abstaining from smoking, at any time point considered (Table 2).

**Table 2:** Efficacy of smoking-cessation therapies in the short term and during follow-up.

	Varenicline	Bupropion	NRT	Bupropion + NRT
Treated, n	225	34	67	11
Abstained from smoking, n (%)				
1 month	170 (76)	30 (88)	47 (70)	10 (91)
3 months	143 (64)	28 (82)	38 (57)	8 (73)
12 months	94 (42)	23 (68)	37 (55)	5 (46)

NRT = nicotine replacement therapy; No significant association between therapy and smoking-cessation success, at each study time point, was found (Cochran-Armitage test for trend in proportions, *P* > 0.05).

### Polymorphisms in CHRNA5 are associated with nicotine dependence

Genotype frequencies of all seven nAChR polymorphisms were found to respect the Hardy-Weinberg equilibrium in our patient series. To test the association of these polymorphisms with nicotine dependence, we considered two nicotine addiction-related phenotypes: CPD and eCO at baseline. Four SNPs in the *CHRNA5* locus (e.g. rs503464, rs55853698, rs55781567 and rs16969968) were found to be significantly associated with both CPD and eCO (Table 3). Of note, the associations of these four SNPs with CPD and eCO remained statistically significant after the correction for multiple testing (FDR < 0.05). No significant association with either eCO or CPD was found for rs3841324 in *CHRNA5*, rs2072661 in *CHRNB2*, or rs2236196 in *CHRNA4*.

**Table 3:** Association between nicotine dependence parameters and SNPs in nicotinic acetylcholine receptor subunit genes, in 337 Italian subjects, by linear regression

Polymorphism	Chr.	Gene^1^	Position (bp)^2^	Minor allele	CPD			eCO		
					β	*P* ^3^	FDR^4^	β	*P* ^3^	FDR^4^
rs2072661	1	*CHRNB2*	154 576 404	T	−0.97	0.30	0.35	0.068	0.95	0.95
rs3841324	15	*CHRNA5*	78 565 480	D^5^	−0.16	0.85	0.85	−0.58	0.55	0.64
rs503464	15	*CHRNA5*	78 565 554	A	−3.5	0.6 × 10^−3^	3.9 × 10^−3^	−3.9	0.80 × 10^−3^	3.7 × 10^−3^
rs55853698	15	*CHRNA5*	78 565 597	G	2.0	8.1 × 10^−3^	0.014	2.8	2.1 × 10^−3^	3.7 × 10^−3^
rs55781567	15	*CHRNA5*	78 565 644	G	2.1	5.6 × 10^−3^	0.013	2.9	1.7 × 10^−3^	3.7 × 10^−3^
rs16969968	15	*CHRNA5*	78 590 583	T	2.2	4.4 × 10^−3^	0.013	2.8	2.1 × 10^−3^	3.7 × 10^−3^
rs2236196	20	*CHRNA4*	63 346 204	C	−1.0	0.19	0.27	−0.95	0.32	0.45

Chr. = chromosome; CPD = cigarettes smoked per day; eCO = expired carbon monoxide.

^1^Gene closest to the SNP or harbouring the SNP.

^2^Genomic position based on Assembly GRCh38.p5.

^3^Linear regression adjusted for sex, and based on additive effects of SNPs, i.e., β > 0 means that there is direct proportionality between the number of minor alleles and the smoking parameter.

^4^False discovery rate obtained using the Benjamini-Hochberg procedure.

^5^rs3841324 (aliases rs67624739 and rs142774214) is a 22-bp insertion/deletion variation, D = deletion.

For the four significantly associated SNPs, linear regression showed two patterns for the impact of the minor allele (Supplementary Figure S1, Table 3). The SNP rs503464 had a negative β value in the association with CPD (*P* = 0.6 × 10^−3^), indicating that as the number of minor alleles (A) increased, the number of cigarettes smoked decreased. On the contrary, the other three significant SNPs (rs55853698, rs55781567 and rs16969968) had β > 0, meaning that an increase in the number of minor alleles was associated with an increase in the number of cigarettes smoked. Regarding eCO, the β values for each significant SNP were concordant with those from the analysis of CPD, meaning that, depending on the number of minor alleles, the variation in eCO goes in the same direction as the variation in the number of cigarettes smoked per day.

### rs503464 in the 5′-UTR of CHRNA5 is associated with smoking cessation

We then evaluated the association of nAChR SNPs with the response to smoking-cessation therapy (Table 4). Only one SNP, rs503464, a variant in the 5′ UTR of *CHRNA5*, was associated with the response (logistic regression, *P* < 0.05). This association was seen at all three follow-up visits, with an OR < 1, meaning that increasing the number of minor alleles (A) confers a lower probability of continuing smoking. This association appears to be free of confounding by nicotine dependence, since baseline eCO was a covariate in the logistic regression. After correction for multiple testing, however, only the association with mid-term response to smoking-cessation therapies remained statistically significant (FDR < 0.05). At the mid-term follow-up, the percentage of patients who abstained from smoking increased progressively with the number of minor alleles at rs503464, so that 10 of the 11 cases with AA genotype (91%) were not smoking at the 3-month visit (Supplementary Figure S2).

**Table 4:** Association between response to smoking-cessation therapies and SNPs in nicotinic acetylcholine receptor subunit genes, at three follow-up time points in 337 Italian subjects, by logistic regression

Polymorphism	Major/Minor allele	MAF	Short-term^1^			Mid-term^1^			Long-term^1^		
			OR (95% CI)	*P* ^2^	FDR^3^	OR (95% CI)	*P* ^2^	FDR^3^	OR (95% CI)	*P* ^2^	FDR^3^
rs2072661	C/T	0.22	0.77 (0.48–1.2)	0.28	0.39	0.68 (0.44–1.1)	0.08	0.14	0.90 (0.61–1.3)	0.59	0.98
rs3841324	I/D^4^	0.33	1.1 (0.73–1.7)	0.67	0.67	1.1 (0.78–1.6)	0.51	0.60	1.4 (0.96–2.0)	0.082	0.22
rs503464	T/A	0.19	0.46 (0.25–0.84)	0.011	0.077	0.46 (0.27–0.78)	4.3 × 10^−3^	0.030	0.59 (0.38–0.92)	0.020	0.14
rs55853698	T/G	0.47	1.4 (0.92–2.0)	0.12	0.26	1.4 (0.97–2.0)	0.077	0.14	1.0 (0.73–1.4)	0.94	0.98
rs55781567	C/G	0.47	1.3 (0.90–1.9)	0.15	0.26	1.3 (0.94–1.9)	0.10	0.14	1.0 (0.73–1.4)	0.98	0.98
rs16969968	C/T	0.47	1.3 (0.91–2.0)	0.14	0.26	1.4 (0.96–2.0)	0.080	0.14	1.1 (0.76–1.5)	0.75	0.98
rs2236196	T/C	0.35	1.1 (0.76–1.7)	0.56	0.65	0.95 (0.66–1.3)	0.75	0.75	0.75 (0.54–1.1)	0.094	0.22

MAF = minor allele frequency; OR = odds ratio; CI = confidential interval.

^1^Time points indicating one month (short-term), three months (mid-term) and twelve months (long-term) after the start of therapy.

^2^Logistic regression adjusted for sex, therapy, and eCO based on additive effects of SNPs, i.e., an OR > 1 means that the risk of continuing smoking increases with the number of minor alleles.

^3^False discovery rate obtained using the Benjamini-Hochberg procedure.

^4^rs3841324 (aliases rs67624739 and rs142774214) is a 22-bp insertion/deletion variation, D = deletion.

**Figure 2:**
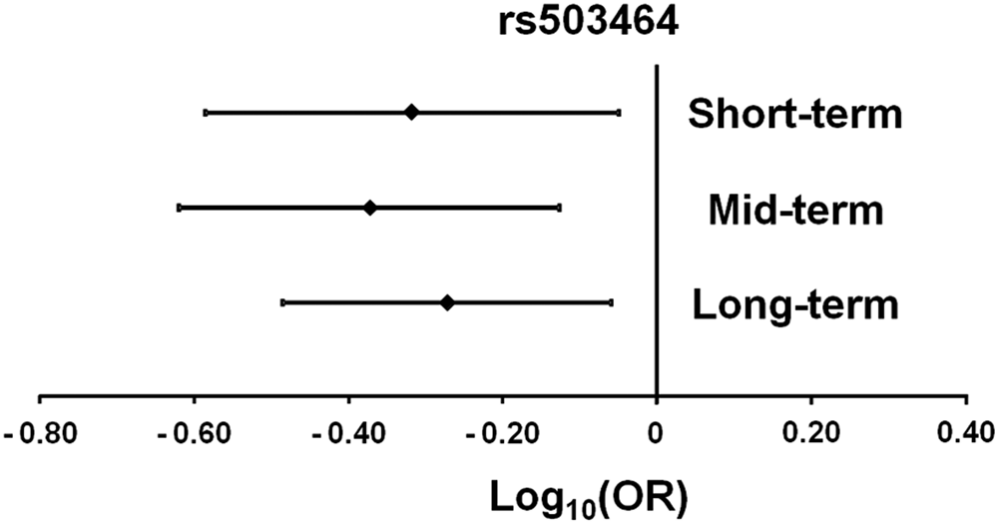
Individuals carrying at least one minor allele (**A**) of rs503464 have a lower probability of continuing smoking than subjects carrying the common allele (OR < 1). Plot of the log-transformed OR (diamond) and 95% confidence intervals at short-, mid- and long-term evaluations after smoking cessation therapies (*P* = 0.020, *P* = 0.0030, and *P* = 0.012, respectively). Logistic regression was carried out using sex and therapy as covariates and considering a dominant model for the minor allele.

We repeated the logistic regression for rs503464 by grouping genotypes according to a dominant model for the minor allele (comparing individuals carrying at least one copy of the minor allele with homozygotes for the common allele). Also with this analysis, the OR for rs503464 was less than one at all three evaluation points, indicating that subjects having at least one minor allele (A) had a lower probability of continuing smoking than subjects carrying the common allele (Fig. 2).

## Discussion

In this study, we tested the involvement of nAChR subunit polymorphisms in nicotine dependence and the response to smoking-cessation therapy, and found an association of *CHRNA5* SNPs with both phenotypes. In particular, the coding SNP rs16969968 and the 5′-UTR SNPs (i.e. rs503464, rs55853698, rs55781567) were significantly associated with both baseline CPD and eCO, taken as measures of nicotine dependence. rs503464 also associated with response to smoking-cessation therapies at all three time points examined. No association with nicotine dependence or smoking-cessation success was found for the two tested SNPs in *CHRNA4* and *CHRNB2*.

Our data are in agreement with the repeatedly reported association of rs16969968 with nicotine dependence^31^ and also confirm the observed association of rs55853698 with smoking quantity^32^. Additionally, we detected a significant association for two 5′-UTR polymorphisms of *CHRNA5* gene (rs503464 and rs55781567) that had never been associated with nicotine dependence. rs503464 was tested for association with nicotine dependence in Bierut *et al*.^24^, but no significant association was found. To the best of our knowledge, rs55781567 has never been reported to associate with nicotine dependence, but only with *CHRNA5* expression levels. Therefore, our results support the influence of individual genetic constitution on smoking intensity. Indeed, both the coding polymorphism rs16969968, altering the protein structure of the α5 subunit, and the regulatory variations upstream of the initiation codon of *CHRNA5* mRNA (rs503464, rs55853698, and rs55781567) were shown here to have a role in the genetic predisposition to nicotine dependence. The latter three SNPs (evaluated as a haplotype together with the promoter ins/del variation rs3841324) were associated with *CHRNA5* expression levels in normal lung tissue^27^ and were also reported to have a functional role in modulating *CHRNA5* promoter transcriptional activity *in vitro* in neuroblastoma cells^25^; these findings suggest that these polymorphisms play a functional role in nicotine dependence by influencing *CHRNA5* mRNA level in the lungs and possibly in the brain.

We found a novel association of rs503464 also with the success of smoking-cessation therapy that was consistent along time and independent of baseline eCO, used as an estimate of nicotine dependence. This result suggests that smoking-cessation phenotype is associated with the alteration of *CHRNA5* mRNA levels, being significantly associated with the regulatory SNP rs503464, rather than with variations in the amino acid sequence of the α5 subunit. Further investigations are needed to understand the mechanism through which the modification of *CHRNA5* mRNA level influences the ability to quit smoking during smoking-cessation therapy. The reported association between the upregulation of brain nAChRs and the success of quitting smoking^33^ supports the hypothesis that modulation of *CHRNA5* levels affects the ability to quit smoking. After multiple testing correction, only for the association of rs503464 with smoking-cessation therapy mid-term response reached statistical significance. Replication of our results in a wider and independent population series would allow to strengthen our findings, also overcoming the lack of information about the ancestry of the genotyped subjects who, however, all had Italian residency.

All four *CHRNA5* regulatory polymorphisms (rs3841324, rs503464, rs55853698, and rs55781567) and the coding SNP rs16969968 that we investigated here have also been associated with lung cancer risk^27,34^. Therefore, the present results support the hypothesis that smoking habit mediates the link between the 15q25 locus and the risk of developing lung cancer^35,36^. Overall, our results strengthen the importance of *CHRNA5* subunit in smoking-related phenotypes.

The assessment of smoking status from the eCO reading is a strength of this study that makes the association analyses more robust, since CPD is a self-reported and, therefore, subjective evaluation^37^, especially when smokers do not admit their failure in quitting smoking. Indeed, it has been reported that eCO is a better biomarker than CPD for use in genetic associations studies^38^.

Collectively, this analysis of genetic variants in nAChR subunits supports the role of individual genetic makeup in the ability to quit smoking. Of note, our findings point to the importance of the *CHRNA5* regulatory SNP rs503464 in both nicotine dependence and smoking-cessation success. Further studies involving larger patient series are needed to validate the association of rs503464 with smoking abstinence during smoking-cessation treatment. Such studies will allow us to demonstrate the clinical utility of this SNP in personalized smoking-cessation therapy, chosen according to the individual genetic constitution.

## Methods

### Study population and clinical database

This study was carried out at the Fondazione IRCCS Istituto Nazionale dei Tumori (Milan, Italy), a public cancer institute. The study protocol was approved by the institute’s Committee for Ethics, and the research was conducted in accordance with the tenets of the Declaration of Helsinki. Overall, 337 adults seeking to quit smoking were studied. Although information on the ethnic origins of these patients was not available, all patients had Italian residency. We included 214 smokers who had received smoking-cessation counselling and treatment at the Tobacco Control Unit of our institution between 2009 and 2012. In addition, we included another 123 subjects who had participated in a smoking-cessation program within the Multicentric Italian Lung Detection (MILD) trial (a pilot observational trial) during 2009–2010 ^39^. Patients had given their informed consent for the collection of personal and clinical information and biological materials for research purposes.

Patients recruited at the Tobacco Control Unit were unselected for age and smoking intensity. According to their smoking characteristics, they had received pharmacotherapy with varenicline, bupropion, NRT alone, or NRT plus bupropion, with various formulations and doses for the durations indicated in Supplementary Table S1. If, after 15 days, the therapy was ineffective or caused side effects, patients could change to another therapy: we considered the first recorded treatment unless one of the treatments was varenicline, in which case we considered this treatment. Patients recruited through MILD were all heavy smokers (defined as > 20 pack-years), were 49–75 years old, and were all treated with varenicline according to the following treatment scheme: 0.5 mg/day for the first three days, 0.5 mg twice daily for following four days, and 1 mg twice daily from day 8 to the end of month 3. All patients included in this study had taken a medication long enough to be considered compliant (Table 5) even if they did not complete the full duration of therapy; this choice was made due to our expectation that a patient’s genetic constitution affects the efficacy of treatment. Patients from both groups (Tobacco Control Unit and MILD) received counselling, as described in Pozzi *et al*.^39^.

**Table 5:** Therapies offered to patients in the study.

Therapy	Duration	Efficacy*	Compliance
Varenicline	3 months	15 days	1 month
Bupropion	2 months	15 days	1 month
NRT	15 days	NA	7 days
Bupropion + NRT	2 months	15 days	1 month

*After this time, patients in treatment at the Tobacco Control Unit could change to another therapy. NA, not applicable.

From the smoking-cessation programs, we obtained data on the patients’ sex, age, and smoking habit at four time points, namely baseline and follow-up at 1, 3, and 12 months after the start of therapy. At each visit, patients self-declared if they were smoking or not, and, if they were still smoking, they self-declared the number of cigarettes per day (CPD) as an indication of smoking intensity. Additionally, they underwent breath testing to measure their exhaled carbon monoxide (eCO), which is produced by the incomplete combustion of carbon-containing material in the lung and provides an estimate of how much smoke has been inhaled. eCO is therefore used to monitor progress in smoking cessation^37^. Baseline values of CPD and eCO were considered to be markers of nicotine dependence. During follow-up, patients were defined as having stopped smoking when they both self-declared smoking cessation and had an eCO reading < 6 ppm. Finally, we obtained a peripheral blood sample that had been collected for genetic studies.

### DNA biobank and genotyping

DNA was extracted from peripheral blood using the DNeasy Blood & Tissue Kit (Qiagen) and was quantified by spectrophotometry (ND-2000c, NanoDrop Products, Wilmington, DE, USA). SNP-containing fragments were PCR-amplified using SNP-specific primers (Supplementary Table S1). Then, six SNPs were genotyped by pyrosequencing: rs2072661 in *CHRNB2* (chr. 1), four SNPs mapping to *CHRNA5* (rs503464, rs55853698, rs55781567 and rs16969968 on chr. 15q25), and rs2236196 mapping in *CHRNA4* (chr. 20q13). Pyrosequencing was performed on a PSQ96MA system (Biotage, Uppsala, Sweden) running PyroMark Q96 ID Software (Qiagen). Additionally, a 22-bp insertion/deletion (ins/del, rs3841324), 71 bp upstream of the *CHRNA5* transcription start site, was genotyped by 3% agarose gel electrophoresis.

### Statistical analysis

The association between therapy and smoking-cessation success, at each study time point, was analyzed with the Cochran-Armitage test for trend in proportions. The congruity of genotype frequencies at each SNP locus was tested with respect to the Hardy-Weinberg equilibrium.

Regression analyses were carried out with data collected at three time points during the treatment protocol. The first time point was one month after the start of therapy and reflects patients’ ability to stop smoking by the fourteenth day of treatment, when treatment efficacy was assessed (short-term response). The second time point was three months after the start of therapy and corresponds to the end of standard therapy (mid-term response). The last time point was 12 months after the start of therapy and indicates if the effects of smoking-cessation therapies can be maintained for a long time (long-term response).

Linear regression was used to assess possible associations between SNP genotypes and both baseline smoking intensity (CPD) and baseline eCO. This statistical analysis was adjusted for sex, and based on the additive effects of SNPs, i.e., β > 0 means that there is a direct proportionality between the number of minor alleles and the smoking parameter. Logistic regression was used to assess associations between SNP genotypes and the response to smoking-cessation therapies (“yes” or “no” for quitting smoking), with sex, therapy, and eCO as covariates. Odds ratios (ORs) were calculated considering an additive effect of SNPs, i.e., an OR > 1 means that the risk of continuing smoking increases with the number of minor alleles. Statistical significance in regression analyses was adjusted for multiple testing using the Benjamini-Hochberg procedure to obtain the false discovery rate (FDR). We also tested a dominant model for the minor allele of rs503464, comparing the group of individuals heterozygous or homozygous for the minor allele with individuals homozygous for the common allele; this logistic regression analysis was adjusted for sex and therapy.

Statistical tests were carried out using PLINK software. Statistical significance was set at *P* < 0.05.

## Electronic supplementary material

Supplementary Information
