# Supplementary material for: Pharmacogenetic study of seven polymorphisms in three nicotinic acetylcholine receptor subunits in smoking-cessation therapies
Source: Sci Rep. 2017 Dec 1;7:16730. doi: 10.1038/s41598-017-16946-6 (PMC5711795; doi:10.1038/s41598-017-16946-6)

Pharmacogenetic study of seven polymorphisms in three nicotinic acetylcholine receptor subunits in smoking-cessation therapies

Giulia Pintarelli [1a], Antonella Galvan [#], Paolo Pozzi [1c ‡], Sara Noci [1a], Giovanna Pasetti [#], Francesca Sala [#], Ugo Pastorino [1b], Roberto Boffi [1c], Francesca Colombo [1a*]

Supplementary information

Supplementary Table 1. Primers for PCR and pyrosequencing

Polymorphism	Gene [a]	Forward primer	Reverse primer	Sequencing primer
rs2072661	*CHRNB2*	BIO-CTGAAGGGAGGGGAAGAGAGA	AAGCAGCGGCAGGTGTCA	GCAGGTGTCAGGTCA
rs3841324 [b]	*CHRNA5*	AAAAGGAACAAGGCGAGGATTG	GAGTGTGAGTCGTGAGACAAAACG	
rs503464	*CHRNA5*			CCAGAAGCTGCTAGG
rs55853698	*CHRNA5*	ACATGCGTCCCGAGCCC	BIO-GCGGGGAACGCGAACTCT	GCGCGGAGCGGCCCC
rs55781567	*CHRNA5*			CGACTCACACTCAGTGC
rs16969968	*CHRNA5*	BIO-CCAAACTGCTTTGCATGAGAA	GGGTCATGCTGTTTACTCTGC	TCTTGTAATGTAGCGAATAG
rs2236196	*CHRNA4*	BIO-CCTGCCTGGACCCTCTCCTAG	GTGTCCTGCGCCTGTGTG	GAGGGTGAGCCAGCA

BIO, modification with biotin
[a] Gene closest to the SNP or harboring the SNP
[b] Aliases rs67624739 and rs142774214; 22-bp ins/del variation (GGGCGGGGCCAGAGGGAAATAG/-), genotyped by PCR and agarose gel electrophoresis

Supplementary Figure S1. Number of cigarettes smoked per day (CPD) and level of exhaled CO (eCO) by genotype, for four SNPs in the *CHRNA5* locus. Dots are mean values and whiskers are SE.

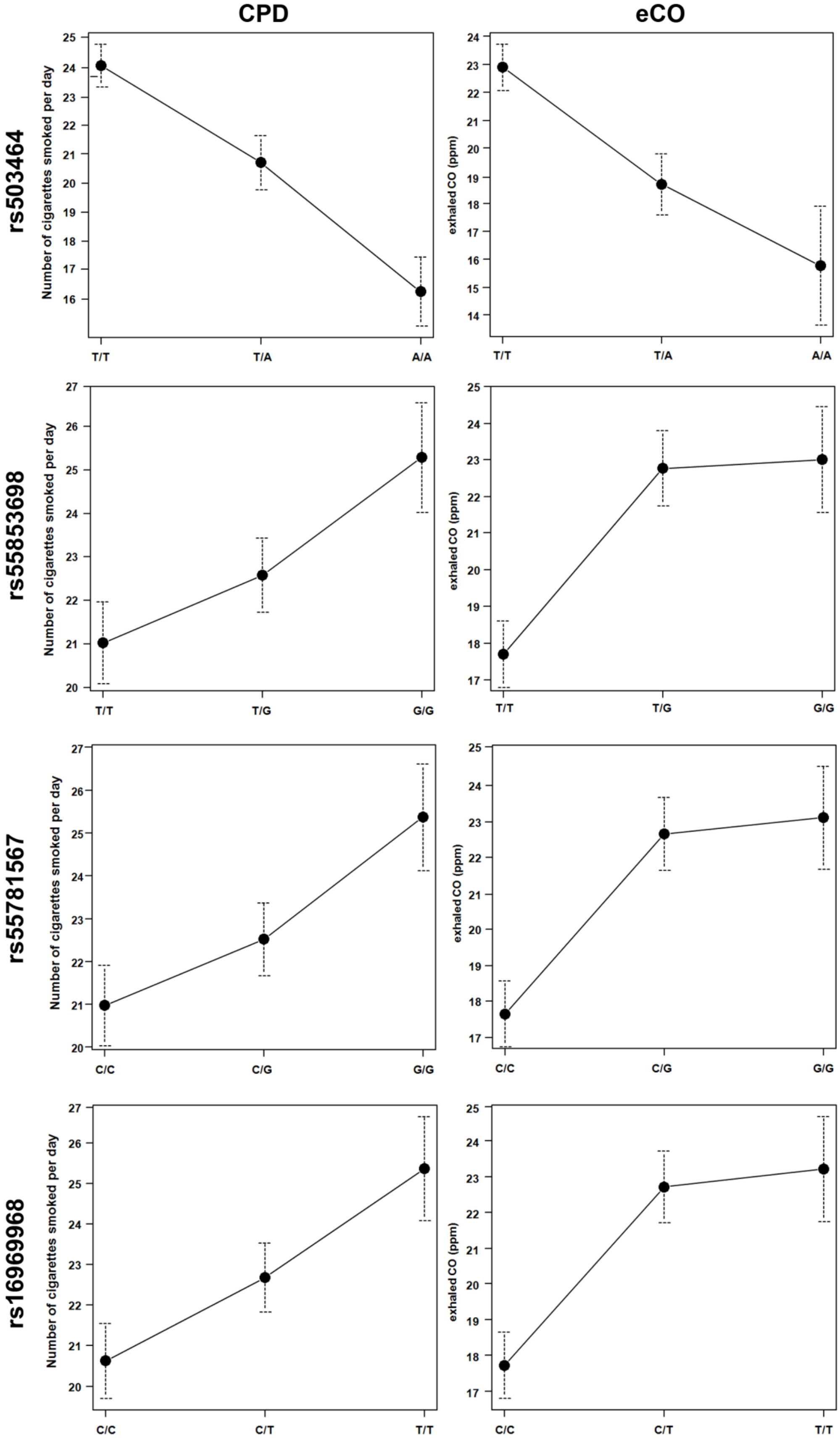

Supplementary Figure S2. Smoking status three months after the start of anti-smoking therapy, by genotype at rs503464 (the number of patients belonging to each genotype group are shown in brackets). Blue bars represent the portion of abstaining patients, whereas the percentage of smoking patients is shown in green.

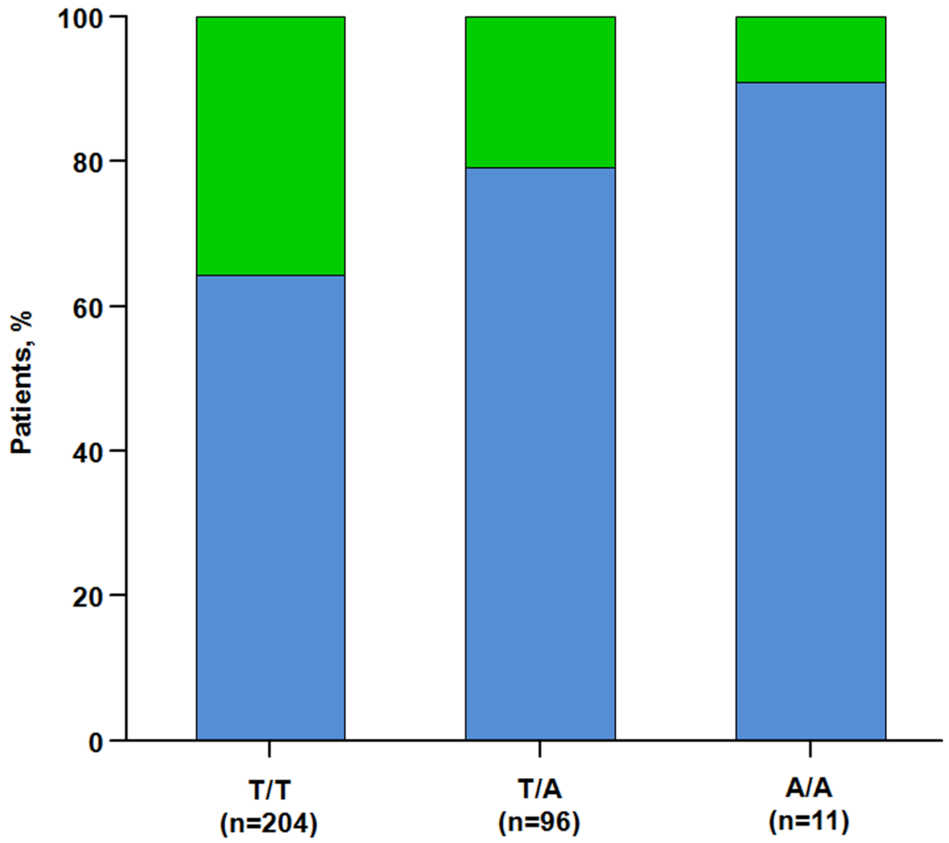

Supplement: Supplementary file 1 — Supplementary Information [file 41598_2017_16946_MOESM1_ESM.pdf]
